# Sound garden: How snakes respond to airborne and groundborne sounds

**DOI:** 10.1371/journal.pone.0281285

**Published:** 2023-02-14

**Authors:** Christina N. Zdenek, Timothy Staples, Chris Hay, Lachlan N. Bourke, Damian Candusso

**Affiliations:** 1 Venom Evolution Lab, School of Biological Sciences, The University of Queensland, St. Lucia, Queensland, Australia; 2 Marine PaleoEcology Lab, The University of Queensland, St. Lucia, Queensland, Australia; 3 Australian Reptile Academy, Brisbane, Queensland, Australia; 4 Queensland University of Technology (QUT), School of Creative Practice, Kelvin Grove, Brisbane, Queensland, Australia; State Museum of Natural History, GERMANY

## Abstract

Evidence suggests that snakes can hear, but how snakes naturally respond to sound is still unclear. We conducted 304 controlled experiment trials on 19 snakes across five genera in a sound-proof room (4.9 x 4.9 m) at 27ºC, observing the effects of three sounds on individual snake behavior, compared to controls. We quantified eight snake behaviors (body movement, body freezing, head-flicks, tongue-flicks, hissing, periscoping, head fixation, lower jaw drop) in response to three sounds, which were filtered pink-noise within the following frequency ranges: 0–150Hz (sound 1, which produced ground vibrations, as measured by an accelerometer), 150–300Hz (sound 2, which did not produced ground vibrations), 300–450Hz (sound 3, which did not produced ground vibrations). All snake responses were strongly genus dependent. Only one genus (*Aspidites*, Woma Pythons) significantly increased their probability of movement in response to sound, but three other genera (*Acanthophis* (Death Adders), *Oxyuranus* (Taipans), and *Pseudonaja* (Brown Snakes)) were more likely to move away from sound, signaling potential avoidance behavior. Taipans significantly increased their likelihood of displaying defensive and cautious behaviors in response to sound, but three of the five genera exhibited significantly different types of behaviors in sound trials compared to the control. Our results highlight potential heritable behavioral responses of snakes to sound, clustered within genera. Our study illustrates the behavioral variability among different snake genera, and across sound frequencies, which contributes to our limited understanding of hearing and behavior in snakes.

## Introduction

While snakes lack external ears and a tympanic middle ear (‘ear drum’), they are not deaf [1]. Hearing in snakes predominantly occurs via sound-induced head vibrations which are received by the quadrate and columella (mammalian homologue of stapes) bones attached to the jawbone. It is thought that, when stimulated by vibration, these bones relay a signal to the cochlear duct through the columella and the perilymphatic fluid of the inner ear [2–5].

Compared to senses such as vision and taste, hearing appears less important to snakes relative to other animals [6]. For example, Pine Snake (*Pituophis melanoleucus*) hatchlings in the laboratory responded more strongly to visual stimuli than vibratory stimuli [6]. However, hearing still may play an important part in snake sensory input and behavior regarding prey acquisition and predator avoidance [7–10]. For example, some evidence supports this hypothesis, particularly in snake avoidance of potentially dangerous prey via sound cues [7, 8]. Sound cues may also be used by snakes alongside sight and smell to accurately strike prey [10]. Finally, hearing in snakes may also provide warning of approaching predators of snake, or larger animals that could trample snakes [6]. In possible support of hearing playing a role in the survival of snakes, Wever [11] describes reptiles (which includes crocodiles, turtles, tuataras, lizards, and snakes) as the most diverse class of vertebrates with regards to their ear structures, suggesting possible selection pressure to evolve structures that suited each taxa.

There is little understanding of the mechanisms of snake hearing. However, evidence suggests their greatest sensitivity to sound lies in low frequencies. For example, *Crotalus atrox* (Rattlesnakes) suspended in a steel mesh basket responded to airborne sounds (emitted from speakers not hard-mounted to the wall but ‘held in position by the surrounding acoustic foam’) between 200 and 400Hz [12]. *Hydrophis stokesii* (Sea Snakes) also exhibited responses to sounds (via an underwater speaker) between 40 and 600Hz, peaking at 60Hz and 500Hz [13], and royal pythons (*Python regius*) had the greatest sensitivity to substrate vibration and sound-pressure at 80–160 Hz [4]. By contrast, human hearing is most sensitive at 2,000–5,000 Hz: more than 10x higher than snakes [14]. A lack of frequency-range sensitivity in snakes has likely resulted from the relaxed selection for phenotypes associated with hearing in all major evolutionary branches of snakes [15].

Snake behavior may vary according to their genus, as explained by the pace-of-life theory. This theory proposes that animals with faster life-history strategies (e.g. quicker growth rate, higher metabolic rate, shorter life span) exhibit higher general activity levels compared to their slow life-history counterparts [16]. While no such study quantifies behavioral responses of different genera of snakes to sound, one study did investigate the anti-predator behavior in 20 species spanning six taxonomic families from Peru; this study found support for behavioral convergence in one family of snakes and their distantly related mimics, despite a great diversity of anti-predator responses observed [9]. However, that study also found that many closely related snakes differed greatly in their anti-predator behavioral responses, suggesting behavior is not necessarily correlated with phylogeny or genetic relatedness.

Most studies investigating hearing in snakes included just one species, and/or either used partially anesthetised snakes or snakes in a hanging basket, both methods of which preclude freely moving responses. Here we study whether behavioral responses of free-moving snakes to three different sounds are divergent across five genera with different life history and hunting strategies: an ambush elapid (family Elapidae) (*Acanthophis*), an active python (family Pythonidae) (*Aspidites*), an arboreal elapid (*Hoplocephalus*) and two active elapids (*Oxyuranus* and *Pseudonaja*). We measured behavioral responses of 19 snakes in a sound-proof room across 304 trials, exposing them to three sets of sound frequencies (0-150Hz, 150-300Hz and 300-450Hz), along with no-sound controls (n = 4 replicates per condition). In particular, we asked: Do sound stimuli influence (1) the likelihood and variety of snake behavioral responses, particularly defensive or cautious behaviors, and (2) the likelihood and direction of snake movement.

## Materials and methods

**Fig 1:**
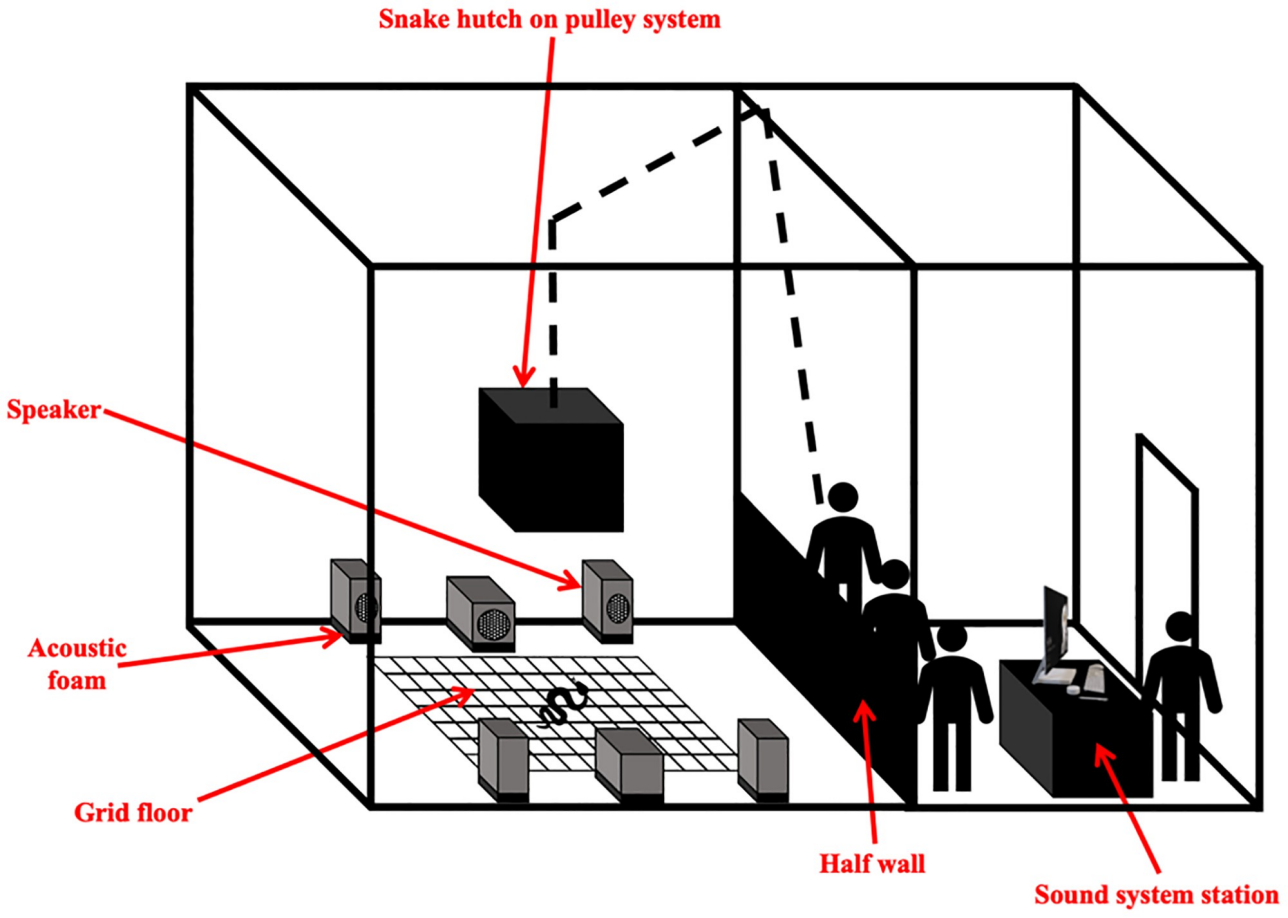
Diagram of sound-proof room for controlled trials. The experimental set-up is shown on the left side, and the data collection area and sound system station are shown on the right side. Image not made to scale. See text for dimensions.

In January 2021, we conducted controlled experiments during the day in a sound-proof room (Fig 1) to investigate the effects of sound on snake behavior and movement.

### The snakes

**Fig 2:**
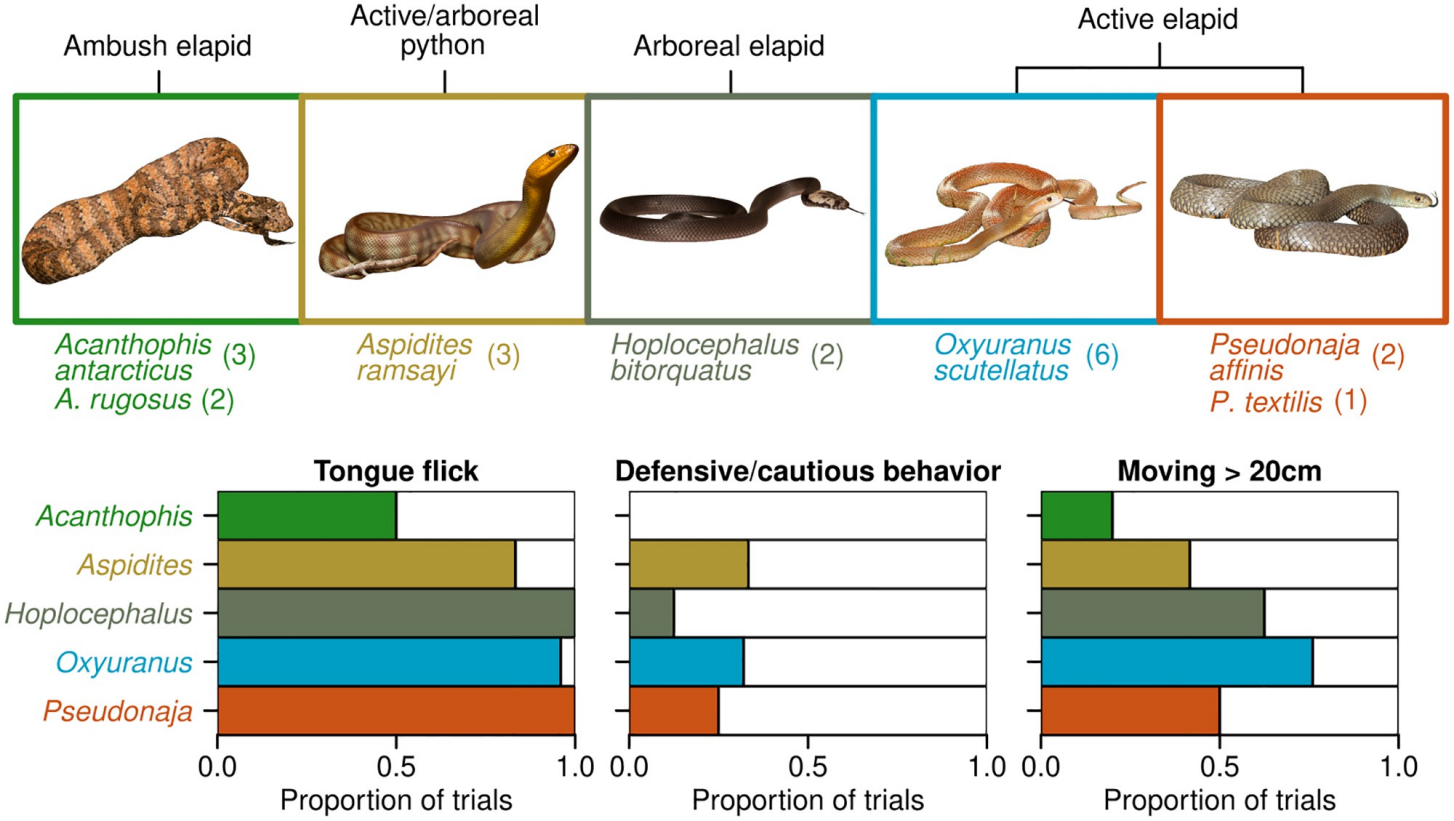
Genus-level behavioral comparisons of snakes in control trials. Genus groupings of snakes, snake count per species (n = 2–6) and presence of three dominant behaviors (tongue flicks, defensive/cautious behaviors and >20cm movement) (from n = 4 trials per snake) as a proportion of control trials (n = 4 per snake). Defensive/cautious behaviors included cautious exploration, fixation, freezing, hisses, head jerks, jaw drops and periscoping (Table 1). Snake images are not to relative scale. See S1 Table in S1 File for the length of all included snakes. Image credits to CNZ: *Acanthophis*, *Aspidites*, *Hoplocephalus*; to CJH: *Oxyuranus*, *Pseudonaja*.

We included 19 captive-bred Australian snakes (adults) representing five genera (*Acanthophis*, *Aspidites*, *Hoplocephalus*, *Oxyuranus*, *Pseudonaja*), ranging in size from 0.35–2.2 m (S1 Table in S1 File; species and sample sizes shown in Fig 2). The snakes ranged in morphological body shapes and foraging types, including active foragers, ambush predators, arboreal species, and constrictor feeders.

**Table 1:** Nine behaviors recorded for snakes included in the study.

Behavior	Description
Directional movement	The distance (cm), direction (toward, away, or orthogonal of the sound), and length of time (sec) in which the snake moves. Measured at the head. Body movement independent of head noted but not measured directly.
Cautionary exploratory movement	The first 1/3^rd^ of body moves in multiple directions while the rest of body is stationary.
Tongue flicks	The number of times the tongue goes in and out of the mouth. One motion of the tongue in and out = 1.
‘Freeze’ response	While snake is alert (head elevated and body tense), a sudden stop of body movement, not in relaxed posture.
Head jerks	Rapid lateral movements of the head independent body movement.
Hisses	Rapid exhalation of air through the mouth to make a hissing noise.
Dropping lower jaw	A distinctive drop of the lower jaw for a period, which exposes the fangs in a defensive manner.
Periscoping	Vertical movement of the head (and sometimes part of body) upwards with a 45º angle or greater, not in an s-curve position.
Fixation	Head facing same direction while body moves laterally.

We used captive (i.e. snakes born in captivity) snakes rather than ‘wild’ snakes because we only had access to captive snakes. Because temperature can substantially affect snake behavior [17–20], our snakes were kept at 27–28ºC prior to testing and during testing. All snake species used are active at these temperatures (CNZ, pers. obs.). Testing occurred in summer (January) during the active period for snakes in the southern hemisphere. We confirmed their body temperature prior to each trial using a remote infrared temperature sensor. Between trials we kept snakes individually in a knotted cloth bag inside a lockable plastic tub with small holes in the lid in a quiet room, also at 27–28ºC. Snakes were provided with water *ad libitum* and maintained on a diet of pre-killed mice prior to the experimental period. All snakes were fed on the same day prior to experiments (10 days prior), and none were fed during the study, ensuring that venom-load-related differences in behavior [21, 22] were eliminated as a confounding variable.

All care, testing, and transport of animals from their permanent residence to the testing location conformed to federal regulations and institutional guidelines for research with venomous snakes and were approved by Biosecurity (Biosecurity QLD Permit #PRID000343) and the QUT Animal Ethics Committee (Approval #2000000816).

### The sounds

Since we included a broader array of species than has previously been tested, we included a broader frequency range than was previously used [4, 12].

The sounds were generated by filtering pink noise using the program Avid Signal Generator. Pink noise is a random signal, filtered to have equal energy per octave. To keep the energy constant over octaves, additional 24 decibels (dB)/octave low-pass and high-pass filters were used. Using pink noise enabled us to cover a range of low frequencies that previous research showed as producing elevated sensitivity in snakes [4].

Filtering via a low-pass and high-pass filter was applied at the following ranges:

- S1: 0-150Hz. A 24db/octave low pass filter was applied at 150 Hz
- S2: 150–300 Hz—A 24db/octave high pass filter was applied at 150 Hz and a 24db/octave low pass filter applied at 300hz
- S3: 300–450 Hz—A 24db/octave high pass filter was applied at 300 Hz and a 24db/octave low pass filter applied at 450hz

We played the aforementioned sounds from three speakers at a time (from one side of the room or the other side, randomly) that were each calibrated to an intensity of 85 dB re 20 uPa SPL, as measured at the centre of the room (1.7m from the speakers). This level is 10–30dB louder than previous work [2, 12] to accommodate the relatively large room and testing area in which we conducted the trials. Loudness of the speakers was calibrated daily using a digital sound meter level (Dick Smith Electronics, Q1362) with a slow mode setting and C-wave weighting for consistent calibrated loudness.

We used an accelerometer placed on the ground to measure any possible ground vibrations produced by the speakers when playing the sounds. Detailed methodology can be found in supplementary text, with results shown in S1, S2 Figs and S6 Table in S1 File.

### The room

Behavioral experiments were conducted in a large (4.9 x 4.9 m) sound-proof room at 27–28ºC, which are temperatures within the snakes’ normal activity range. The testing area (2.4 x 2.4 m) within the large room enabled free movement of even our largest snake (2.2 m total body length) across the timber floor (6mm thick Masonite, supported by plywood). The room was set up as two mirrored halves, with three speakers on each halve, totalling six speakers on the floor surrounding and facing the snake hutch (60 x 60 x 40 (w x l x h)) in the middle of the room (Fig 1). Each side of the room had two full-range speakers on either side of one sub-woofer speaker (Rokit 10s, KRK Systems). All speakers were buffered at their base with a 3cm layer of dense acoustic foam (Auralex Acoustic Foam) in an attempt to limit vibrations from transmitting through the floor (although this had no effect; see results in S1 Fig and S6 Table in S1 File). The speakers were 1.7 m away from the centre point of the room, and the edge of the snake hutch was placed at a consistent 1.2 m from the speakers for each trial.

### The trials

Trials were conducted in January, which is an active-period month for most Australian snakes that have been studied [23–25]. All trials were conducted during daytime because all the included snake species are either diurnal or known to be occasionally active diurnally. A 10%-bleach cleaning solution was used to clean the floor between trials. Prior to beginning experimental trials, one snake at a time was given one 5-minute period of familiarization to freely explore the room.

Trials included a 30 second settling period for the snake inside a custom-built snake hutch (bottomless timber box attached to a pulley system overhead), then the hutch was remotely raised to reveal the snake in the testing arena, where they were presented with either a 30 second period of sound or no sound. We randomized the side of the room that the sound played from during each trial, and we randomized the order of snakes per block (replicate) of treatment. Across the study each snake was exposed to four replicates each of three sounds and the negative control (no sound). For each period and trial, we quantified the response variables listed in Table 1, some of which were adapted from Young & Aguiar (2002) [12].

Due to various logistical constraints, data collection was collected live and in a ‘non-blind’ fashion: that is, the observers knew whether sound was playing or not [26]. All behaviors were simple to measure and not at risk of bias, requiring little to no interpretation. That is, objective descriptors (Table 1) left little capacity for alternate interpretations. Only one behavior (body movement) was a continuous variable, which was cross-checked via video review immediately after each trial.

### Statistical analysis

All data processing and statistical analyses were conducted in R version 4.02 [27].

### Summarizing snake behavioral responses

Defensive and cautious behaviors included six distinct behaviors: freezing, hissing, fixation, head jerks, lower jaw drops, periscoping and cautionary exploration (Table 1). We converted the sum of these for each trial into a binary variable, whether at least one defensive/cautious behavior occurred. This binary variable was modeled as a response variable in one of the probability models.

Many snakes recorded small head movements during trials. We summed head movement in all directions to reflect the magnitude of movement. We observed a natural division between snakes with < 20cm head movement and those with substantially larger movement. We divided movement into less than and greater than 20 cm binary categories for probability modeling.

Movement response does not resolve whether snakes were moving towards or away from the sound, which has different biological implications. As such, we constructed an additional model only for snake trials with >20 cm head movement. We constructed a binary variable, with all trials with the greatest magnitude of head movement occurring away from the speaker as successes, and with all other trials treated as failures. This model estimated the probability that snakes moved away from the speaker.

### Sound treatment models

We tested for an effect of sound treatment using Bayesian hierarchical models (brm function, brms package [28]⁠, fit with two interacting fixed effects: sound treatment (four level factor) and snake genus (five level factor). Repeated measurements made on individual snakes were nested within a snake-level random intercept term. This allowed for variation from individual snakes to be estimated and to appropriately account for multiple dependent measurements taken from the same animal. We excluded *Acanthophis* and *Hoplocephalus* trials from the defensive/cautious behavior model due to their lack of non-zero trials.

We specified a Bernoulli likelihood function with vague uninformative priors for intercept and slope terms (normal distribution with μ = 0, σ = 5). We specified random intercepts for each snake, each block of trials and the two speaker directions, with cauchy priors (μ = 0, σ = 5). Models were run across four chains for 10,000 iterations each, 5,000 warm-up and 5,000 for sampling, for a total of 20,000 sampling iterations. We set adapt delta to 0.999 and max treedepth to 15 to reduce divergent transitions. Chain convergence in models was evaluated via R-hat scores. Model validation was performed via residual simulation (DHARMa package, [29]⁠), including model uniformity and dispersion tests, and leave-one-out cross validation (loo function, loo package, [30]).

We visualized snake response to control conditions as the mean posterior probabilities made at the population scale (i.e. ignoring random effects), with corresponding 95% credible intervals. To estimate the response difference between control and treatments, we subtracted each genus’ control posterior draws from the posterior draws from each respective sound treatment. This put each genus on an equivalent ‘difference from control’ scale. The means of these resulting distributions reflect mean difference from control to treatment; whereby if the 95% credible intervals of these differences do not cross zero, it suggests evidence of a substantial difference in response probability between the control and treatments.

Two models initially included additional variables; the defensive/cautious behavior model initially included snake sex and movement away from speaker model initially included initial head direction. These additional variables did not improve model fit, as determined via comparison of leave-one-out cross-validation information criteria (LOOIC), interpretable as per AIC. Defensive/cautious models with and without snake sex had LOOIC scores of 223.9 and 223.5 respectively, and movement away from speaker models with and without initial head direction had LOOIC scores of 378.8 and 372.9 respectively.

### nMDS of defensive/cautious behaviors

We explored whether the composition of defensive/cautious behaviors changed based on sound treatment and genus identity. We summed all defensive behaviors for all snakes of a given genus for each sound treatment. We converted these to relative abundance measures (dividing by the total sum of behaviors in that genus-sound treatment combination). These formed the rows of a compositional matrix, with behaviors as columns. We visualized differences in composition using non-metric multidimensional scaling (metaMDS function, vegan package [31]), using Bray-Curtis dissimilarity. We also tested for whether genus or sound treatment significantly affected composition of defensive/cautious behaviors using a PERMANOVA with 999 permutations (adonis2 function, vegan package [31]).

## Results

We conducted 304 trials testing the effect of sound on snake behavior and movement.

### Genus-based responses

We observed substantial inter-genus differences in our target behaviors: tongue flicks, head movement, and a collection of behaviors grouped as ‘defensive or cautious’ (‘Freeze’ response, head jerks, hisses, dropping lower jaw, periscoping and fixation behavior) (Fig 1, Table 1). Trials in all genera except *Acanthophis* contained a close to 100% tongue-flick response (Fig 2). Exhibiting defensive and cautious behaviors was less common, with genera likely to exhibit a target behavior in 20–30% of control trials, with the exception of *Hoplocephalus* (15%) and *Acanthophis* (0%) (Fig 2). Substantial head movement was greatest in *Oxyuranus* snakes, although was observed in at least *c*. 50% of trials for all genera except *Acanthophis* (Fig 2).

### Defensive/cautious behaviors

**Fig 3:**
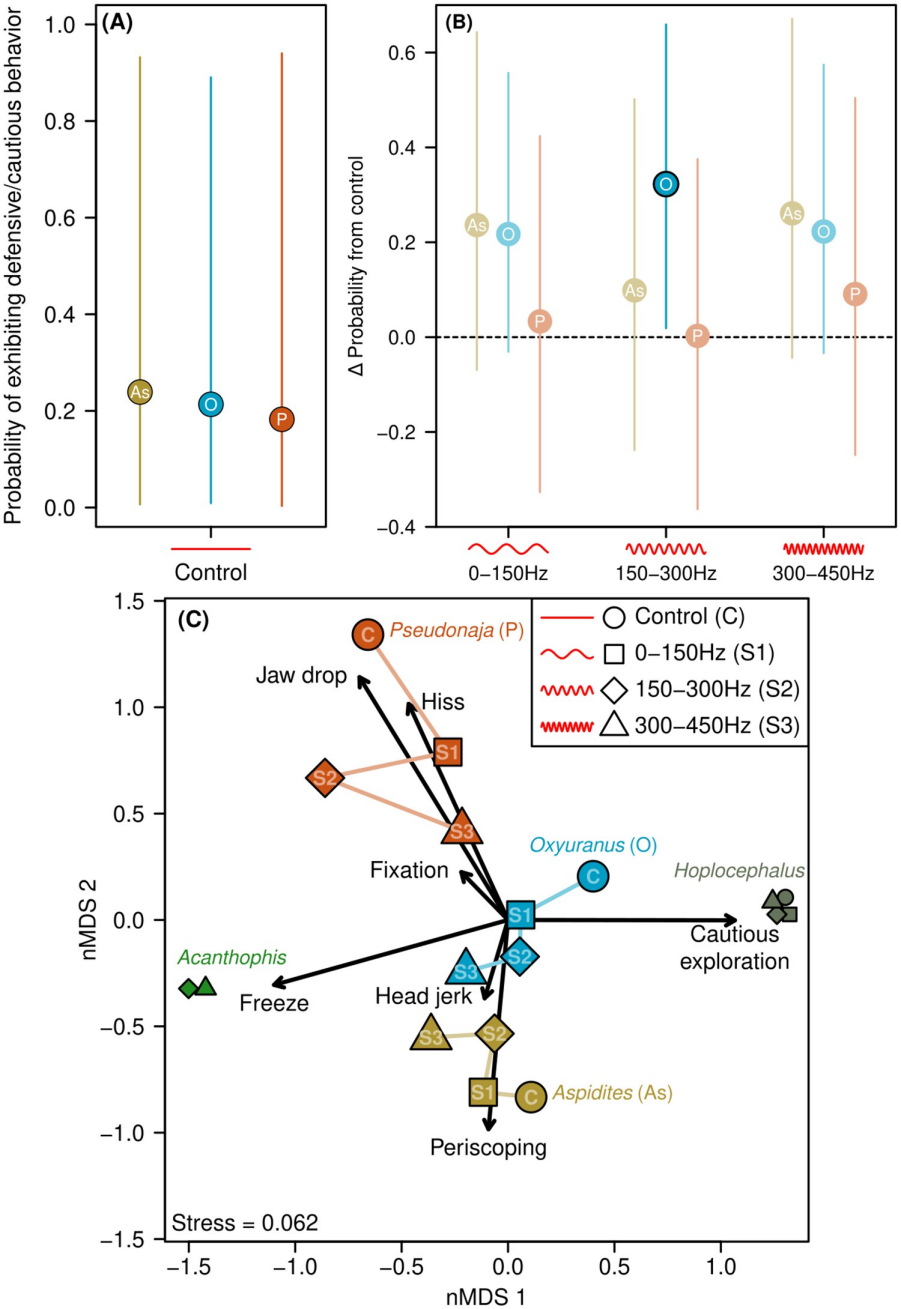
Defensive/cautious behavior responses of 19 snakes to sound compared to controls. **(A)** Mean probability of exhibiting defensive or cautious behavior in control trials for three snake genera with sufficient response (Fig 2). Behaviors included cautious exploration, fixation, freezing, hisses, head jerks, jaw drops and periscoping from n = 4 trials per snake. Lines represent 95% credible intervals. **(B)** Change in probability of exhibiting defensive or cautious behaviors from the control to each of three sound treatments. Values above zero indicate increase in probability, and vice versa. Faded points had credible intervals crossing zero, suggesting no change in probability from control (n = 4 per snake). **(C)** Non-metric multidimensional scaling of defensive/cautious behavior composition for each genus-sound treatment combination. Points with similar behavior composition are clustered on the plot, and are sized based on the total count of behaviors. Points in the same direction as arrows exhibited more of those behaviors, with arrow length proportional to strength of influence. Differences between control (“C”) and treatment points (“S1”, “S2” and “S3”) suggest the type of exhibited defensive behaviors changed in response to sound treatments. Symbols for the control and S1 are omitted for Acanthophis due to no behaviors being exhibited in those trials. Numbers are omitted from Acanthophis and Hoplocephalus symbols due to space constraints.

We first modelled the probability of defensive and cautious behaviors as a function of genus identity and sound treatment, accounting for individual snake variation, trial blocks and speaker arrangement via random effects. Bayesian R^2^ scores for this model were 0.206 (marginal, fixed effects only: 95% CIs 0.084–0.317) and 0.425 (conditional, fixed and random effects: 95% CIs 0.372–0.475), suggesting random effects were equally important as genus identity and sound treatment factors in explaining the likelihood of defensive or cautious behaviors (S3 Table in S1 File). Estimates of the standard deviation of random effects were 2.90 for snake identity, 1.04 for speaker location and 0.71 for block of trials. This suggests most of the conditional variance was due to between-snake differences in the likelihood of exhibiting a defensive or cautious behavior, which captures variation due to snake sex or age. This within-snake variation contributed to results with wide credible intervals of population predictions (Fig 3A and 3B). Even so, we observed an increase in the likelihood of defensive or cautious behaviors in *Oxyuranus* in all three sound treatments (relative to control), and a lesser effect with *Aspidites* in the S1 and S3 treatment (Fig 3B).

**Fig 4:**
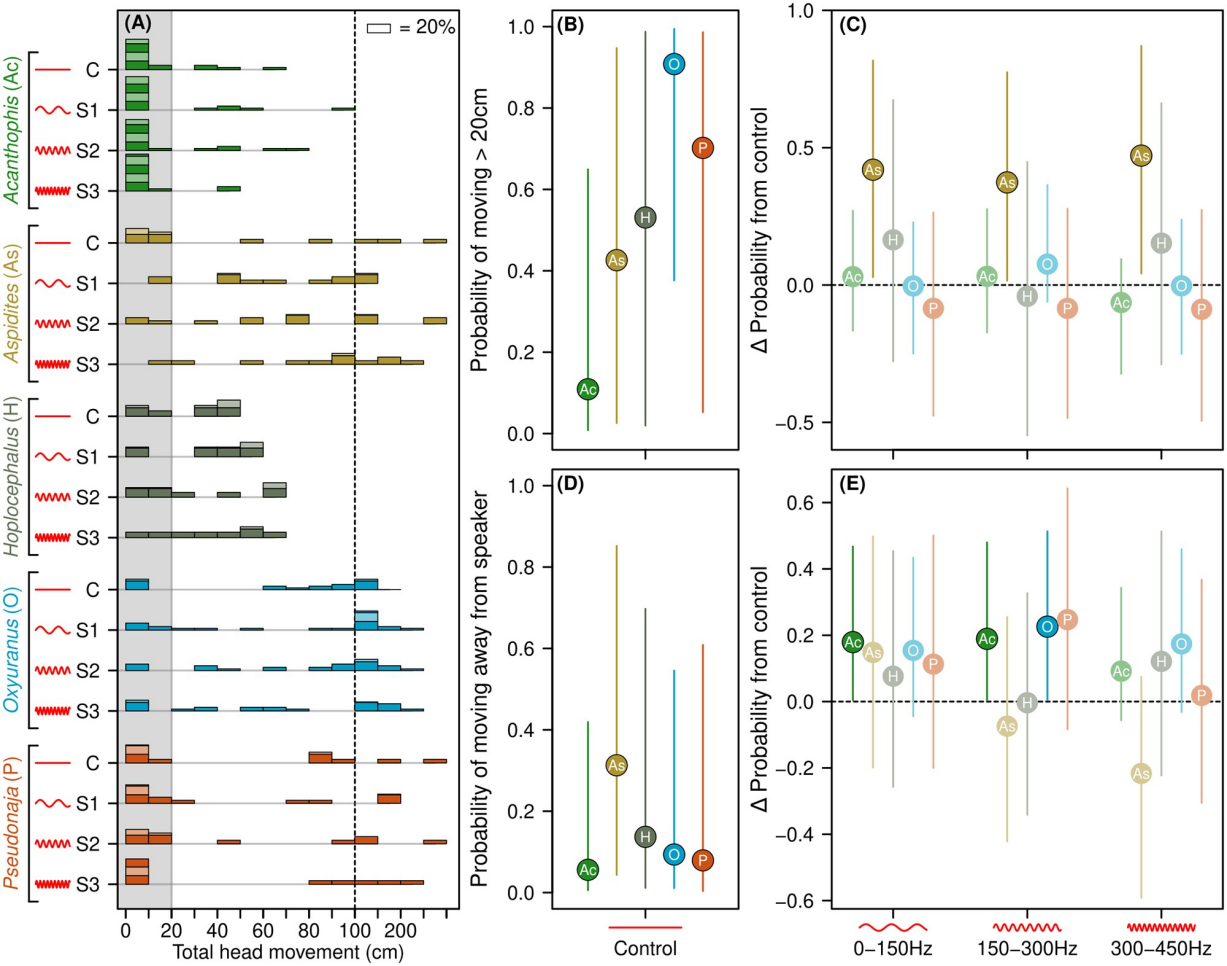
Snake movement in response to sound compared to controls. **(A)** Histogram of snake movement by genus (colored groupings) and sound treatment (C, S1-S3) (n = 4 per treatment per snake). Histogram bars are grouped in units of 10cm, except above 100cm (right of dashed line) where they are grouped in units of 50cm. Alternating colors on bars represent blocks of up to 20% of trials. Grey shading indicates the cut-off used to distinguish 20cm threshold for binary movement response used in probability model. **(B**) Mean probability of >20cm snake movement in control (n = 4 per snake). **(C)** Change in probability of movement from the control to each of three sound treatments. Values above zero (dashed line) indicate increase in probability, and vice versa. **(D)** Mean probability of movement away from speaker for each genus in control trials, using only snakes with >20cm of movement. **(E)** Change in probability of movement away from speaker compared to control means for each of three sound treatments, as per (B). Lines in (B-E) represent 95% credible intervals from Bayesian hierarchical models. Points in C and E with 95% credible intervals that do not cross zero are highlighted as significant effects.

While the probability of observing defensive or cautious behaviors was not strongly linked to sound treatment, especially in *Pseudonaja*, we observed differences in the composition of these behaviors across each genus and between sound treatments (Fig 4C). We found that genus identity explained 88.91% of the variation in behavior composition (F_4,17_ = 36.279, *P* = 0.001), with sound treatment contributing an additional 3.605% (F_1,17_ = 5.884, *P* = 0.001), and no significant variation explained by their interaction (2.580%: F_4,17_ = 1.053, *P* = 0.421). All three genera in the probability model (*Aspidites*, *Oxyuranus* and *Pseudonaja*) were defined by different sets of behaviors in the control trials, with behavior composition changing across the three sound treatments. As sound frequency increased, *Aspidites* became more likely to freeze and less likely to periscope, *Oxyuranus* increased in freezing, head jerks and was less likely to hiss and cautiously explore, and *Pseudonaja* became less likely to hiss and more likely to freeze, head jerk and periscope (Fig 3C). Behavior composition became more similar between the genera as sound frequency increased; Bray-Curtis dissimilarities between control trials for these three genera were *c*. 2x larger than behavior composition in the S3 sound treatment (*Aspidites-Oxyuranus*, 0.657 vs 0.298; *Aspidites-Pseudonaja*, 0.786 vs 0.444; *Oxyuranus-Pseudonaja*, 1.000 vs 0.576).

### Snake movement

Snake movement patterns varied by genus. *Acanthophis* was the least likely to move and *Hoplocephalus*, while likely to move, rarely moved more than 50cm from starting position (Fig 4A). The other three genera exhibited a range of movement, with *Aspidites* showing uniform patterns of movement between 0cm and 1m, and *Oxyuranus* and especially *Pseudonaja* likely to either stay still or move a substantial distance (Fig 4A).

The probability of head movement greater than 20cm also varied by genera. *Acanthophis* was the least likely to move in control trials, *Oxyuranus* the most, and the other three genera varied strongly across individual snakes (Fig 4B). Marginal and conditional R^2^ values were 0.365 (95% CIs = 0.256–0.454) and 0.504 (CIs = 0.469–0.538) respectively. As with the probability model of defensive and cautious behaviors, most random variation was explained by differences between individual snakes (SD = 2.04), compared with trial blocks (SD = 0.23) and speaker sides (SD = 0.92).

In the three sound treatments, we observed a consistent increase in the probability that *Aspidites* moved compared with controls, especially in S3, where almost all trials resulted in movement (Fig 4A and 4C). Other genera showed no response in the probability of movement (S4 Table in S1 File).

When considering the direction of movement, all genera showed a low likelihood of movement away from the speaker (S5 Table in S1 File), consistent with random choice of direction (*c*. 25%). We found *Acanthophis* was more likely to move away from the sound in S1 and S2 treatments, and *Oxyuranus* was more likely to move away in S2, with slightly lower probabilities in S1 and S3 (Fig 4D). *Pseudonaja* showed a weak propensity towards greater probability of movement away from the sound source in treatment S2, but not in S1 and S3. While no *Aspidites* differences were likely to be non-zero, we observed a trend from movement away from the sound source in treatment S1 to a trend to move towards the sound source in treatment S3.

## Discussion

Our study quantified the behavior of free-moving snakes in response to three sounds: one of which produced ground vibrations (S1) and two of which did not and were thus airborne sounds (S2 and S3). We included 19 captive snakes across five genera, from six species, ranging in size (0.35–2.2 m), and covering multiple foraging modes, body morphologies, and venom presence (venomous and non-venomous). We found the behavioral response of snakes to sound differed based on sound frequency and was strongly linked to genus identity. The similar proportion of variation in behavior explained by genus identity and sound frequency compared to that of within-snake clustering suggests that snake behavioral responses are a combination of heritable and environmental components.

The composition of defensive and cautious behaviors exhibited across all trials (sound and no sound) were strongly genus dependent (Fig 3; S2 Table in S1 File). *Oxyuranus* exhibited all behaviors except for periscoping. *Acanthophis* and *Hoplocephalus* responses were dominated by freeze and cautious exploration responses, respectively, with essentially no other behaviors exhibited. Being ambush predators, *Acanthophis* snakes have low activity levels, so a freeze response to potential danger—in combination with physical (and possibly chemical [32]) camouflage—may help them escape detection. *Acanthophis* snakes significantly moved away from the speaker during trials with S1 and S2. While the S1 produced measurable ground vibrations (S1 Fig and S6 Table in S1 File), S2 did not. It is possible that the lower jaw profile of these snakes, which is naturally flattened toward the ground, give them relatively superior tactile sensitivity to ground vibrations (that are transmitted through to the inner ear), which may have enabled them to sense ground vibrations from S2 that our accelerometer could not measure. In addition, behavioral responses in these less active snakes may be subtle, and potentially detectable via physiological responses such as heart rate or hormone levels [33, 34], measuring heart rate would likely prohibit the freedom of snake movement that our present paper achieved.

The genus *Pseudonaja* was included in the present study due to their frequent conflict with humans [35], their reputation as an irritable species [36], and the presence of overt defensive behaviors (e.g. maintained elevated coiling). Interestingly, *Pseudonaja* snakes exhibited a largely dichotomous response to sound, whereby they either remained still or moved a large distance (Fig 4A). This ‘all or nothing’ response aligns with previous work: when humans approached wild Eastern Brown Snakes (*P*. *textilis*) in paddocks/fields, about half of the time the snakes retreated and on most other occasions the snakes relied on crypsis (staying still) [23]. The broad alignment in the behaviors of our captive *Pseudonaja* snakes to those in the wild suggest that the behavioral response of this genus—and perhaps many or all other snake genera—is an innate response rather than a learned response. Likewise, newly hatched and previously unfed Hognose Snakes (*Heterodon platirhinos*) exhibited the typical hognose feign response (inverted body, mouth open, motionless) to predators (humans and owls) [37]. And similarly, 21 species within Crotalinae (Rattlesnakes) responded to scents of snake-eating snakes by exhibiting the same behavior of body bridging (ie. elevating the middle portion of the body to form a bend) [38]. In contrast, a study of 20 species of Peruvian snakes revealed that behavioral responses to predator simulations were more convergent based on mimicry (e.g. aposematic markings) rather than phylogeny or genetic relatedness [9]. This suggests that behavioral mimicry is tightly linked with colour pattern convergence (mimicry of aposematic coloration) and that phylogenetic relatedness may not be the sole predictor of behavioral responses to stimuli.

Very few studies have confirmed the existence of heritable behavior traits in snakes [39, 40], compared to many such studies on mammals, amphibians, birds, and insects (for summary, see Bell et al. 2009). In general, however, certain behaviors (e.g. temperament, courtship) are more repeatable and heritable than others (e.g. mate preference) [41]. We found *Pseudonaja* responses to sound were dominated by jaw drops (gaping) and hisses, whereas *Aspidites* exhibited mostly periscoping and head jerk behavior. Genus-level consistency in these behaviors suggest they are inheritable, i.e. a pre-programmed response to certain stimuli. Defensive behaviors in particular in snakes may be genetically inherited due to many predation pressures upon snakes. We herein coin the term ‘Inherited Defensive Responses’ to refer to the typical and shared defensive behaviors shared by individuals within the same species or genus, which is likely linked to the species ‘pace-of-life’ (see below). Indeed, the fact that multiple individuals within each genus in the present study exhibited a similar suite of behaviors to each other which was different from other genera suggests that the traits are likely heritable in these species and increase survivorship within its specific niche [42, 43]. A study of 172 laboratory-born Garter Snakes (*Thamnophis radix*) in Illinois, USA found a variety of antipredator behaviors within the population, yet the behavior of individuals was consistent [39], suggesting that these behavioral differences are in part heritable. Future work should investigate the heritability of individual-specific and genus-specific behaviors in snakes.

The life-history strategies and behavior exhibited by an animal—and thus its personality—may be largely determined by its ‘pace-of-life’. In brief, this idea links activity levels of a species with its life-history strategies, as explained previously. For example, a study on multiple Garter Snake (*Thamnophis*) species that differed in pace-of-life found that snakes from a fast-living ecotype were more active, as measured by tongue flicks and movement, with the converse being true for slow-living ecotype snakes [40]. We observed a similar pace-of-life pattern, with the ambush predator genus (*Acanthophis*) exhibiting the fewest tongue flicks and movement overall (Fig 2).

Beyond pace-of-life, we observed evidence of snakes orienting based on the source of the sound. *Aspidites* (Woma Pythons) exhibited a trend away from the sound source in treatment S1 and a trend to move towards the sound source in treatment S3. In contrast, three other genera (*Acanthophis*, *Oxyuranus*, and *Pseudonaja*) were more likely to move away from the source of sound, suggesting potential avoidance behavior. These contrasting responses may be explained by a difference in the number of predators of these snakes, and therefore their nervous disposition. *Aspidites* are large (up to 2.7m and 5kg) pythons that prey largely upon monitor lizards (*Varanus gouldii*) and are mostly active nocturnally [25], when most raptors are not active. On the other hand, adult snakes within the genera *Acanthophis*, *Oxyuranus*, and *Pseudonaja* are much smaller in weight (40g–2kg), either partially or mostly diurnal, with many predators such as monitor lizards, raptors, and feral cats [44]. These results differ from Young and Aguiar [12], which found no acoustic orientation in an ambush predator (Western Diamondback Rattlesnake *Crotalus atrox*), yet similar to Young and Morain [10] who observed an ambush viper *Cerastes cerastes* localise small, free-moving mice spatially using groundborne vibrations. Thus, while animals with external and middle (tympanic) ears can hear a greater range of frequencies (10Hz–100kHz) [45] than can snakes (<1kHz) [4], with about 40dB increase in hearing sensitivity [4], snakes are still able to orient with respect to sound.

In our study, the types of behaviors exhibited by the snakes depended on the presence of sound and the type of sound (S1, S2, and S3). Like Young & Anguiar [12], we observed fewer tongue flicks overall in response to sound. One genus (*Oxyuranus*) significantly increased their likelihood of displaying defensive or cautious behaviors in response to sound (only to S2), suggesting an awareness and fear of that particular sound. Snakes in this genus are active foragers, travelling to find and pursue their prey; this likely results in greater risk of—and therefore trepidation for—encountering predators. Unfortunately, comparison of S2 (150–300Hz) to the frequencies of non-vocal sounds emitted by approaching predators (across various habitats) is unknown.

Further evidence of perceptive distinction of different sound types was the change in behavior(s) with increasing sound frequency. *Aspidites* became more likely to freeze and less likely to periscope, *Oxyuranus* increased in freeze behavior, head jerks and was less likely to hiss and cautiously explore, and *Pseudonaja* became less likely to hiss and more likely to freeze, periscope, and head jerk. In addition, the behavior difference between genera in S3 was c. 50% of that in controls, highlighting a potential convergent behavioral response of snakes to sound across both elapid and python species. This potential convergent response may indicate the presence of an archetypal response to sound that evolved prior to splitting of the two families (Elapidae and Pythonidae) around 86 million years ago (median of 64.6–93.5 range from 24 citations) [46] and may dominate the genus-based differences discussed earlier. Christensen et al. [4] reported significant differences (head vibrations of partially anaesthetized snakes) at multiple frequencies between 80Hz and 500 Hz, with peak sensitivity at 160Hz, but no other behaviors were able to be measured. Our results provide some behavior-based evidence of perceptive distinction of different sounds by snakes.

The use of captive snakes instead of wild snakes resulted in the length of captivity being positively correlated with the age of our snakes. As such, we were unable to discern whether age or length of time in captivity was responsible for the lack of responsiveness to sound exhibited by older individuals. While there is unlikely to be substantial genetic differences between captive and wild snakes, their behavior may differ, with this difference possibly becoming more pronounced with longer duration in captivity due to their longer exposure and possible habituation to many sounds (e.g. vacuum, voices, music). However, if snakes are mostly driven by innate, instinctual behaviors, and since there is no biological suggestion that the genes underpinning behavior can change in captivity over the course of an individual’s life, our results may be applicable to wild snakes. Regardless, future research should conduct similar controlled experiments to test the behavioral response of wild snakes to sound, including ultrasonic snake repellent devices embedded into the ground. Future work should also investigate how head size, shape (e.g. surface area of jaw touching the ground), and form (e.g. amount of fatty tissue density) affects sensitivity to sound.

Snake behavior is depauperate in the literature relative to other taxa (e.g., birds, mammals). The common perception of snakes being deaf likely derives from a combination of 1) snakes’ limited hearing ability (regarding frequency range and sensitivity), and 2) peoples’ limited ability to notice and interpret many subtle snake behaviors. To our knowledge, our study is the first to test the free-moving responses of snakes to sound and thus provides a framework for similar future work. Our data reveals that Australian snakes respond to airborne and groundborne sounds, at least within the frequency range of 0–450Hz, and that behavioral responses differ significantly according to genus. Our results improve our limited understanding of snake behavior, which may help humans deter snakes and/or avoid snakebite.

## Supporting information

S1 File (DOCX)
