# Correction: Sound garden: How snakes respond to airborne and groundborne sounds

**DOI:** 10.1371/journal.pone.0321508

**Published:** 2025-04-01

**Authors:** Christina N. Zdenek, Timothy Staples, Chris Hay, Lachlan A. Bourke, Damian Candusso

The fourth author’s name is spelled incorrectly. The correct name is: Lachlan A. Bourke. The correct citation is: Zdenek CN, Staples T, Hay C, Bourke LA, Candusso D (2023) Sound garden: How snakes respond to airborne and groundborne sounds. PLoS ONE 18(2): e0281285. https://doi.org/10.1371/journal.pone.0281285.
