# Supplementary material for: Sound garden: How snakes respond to airborne and groundborne sounds
Source: PLoS One. 2023 Feb 14;18(2):e0281285. doi: 10.1371/journal.pone.0281285 (PMC9928108; doi:10.1371/journal.pone.0281285)
Supplement: S1 File — (DOCX) [file pone.0281285.s001.docx]

Supplementary Materials for

**Sound Garden: How snakes respond to airborne sounds**

Christina N. Zdenek*, Chris Hay, Timothy Staples, Lachlan Bourke, Damian Candusso

*Corresponding author. Email: [ChristinaZdenek@gmail.com](mailto:ChristinaZdenek@gmail.com)

**This PDF file includes:**

Figures S1–S2

Supplemental methods

Table S1–S5

Movie S1 (link only)

**Other Supplementary Materials for this manuscript include the following:**

Movie S1 (actual 38MB file)

Figure S1. Floor vibration measured as absolute delta gravitational acceleration from the mean of no sound controls, as a function of three sound frequencies and three insulation treatments (“-“ = no insulation, “F” = foam under speaker, “H” = speaker held by technician). Black points and lines are mean and 95% credible intervals obtained from a hierarchical Bayesian model. Grey points are individual measurements obtained at 30Hz. Measurements were taken at three distances from the speaker, but as distance was not significant (Table 1), are shown in aggregate here. Several measurements in the 0-150Hz no insulation and foam treatments are beyond the y-axis limit (n=3, to a maximum of 0.170).

Figure S2. Floor vibration measured as absolute delta gravitational acceleration from the mean of no sound controls, with the 0-150Hz results across three insulation treatments (“-“ = no insulation, “F” = foam under speaker, “H” = speaker held by technician) compared with a positive control: a technician jumping immediately adjacent to the phone. Black points and lines are mean and 95% credible intervals obtained from a hierarchical Bayesian model. Grey points are individual measurements obtained at 30Hz. Measurements were taken at three distances from the speaker, but as distance was not significant (Table 1), are shown in aggregate here. Several measurements in the Jump positive control were beyond the y-axis limit (n = 7, max of 7.810).

Supplemental Methods: Accelerometer data

Accelerometer data were collected using the “Accelerometer” iPhone app by DreamArc. The use of an iPhone as an accelerometer has been proven accurate for scientific purposes multiple times [1,2]. Measurements were taken at 30Hz with the phone lying screen up, with three crossed variables: three insulation treatments (none, insulating foam under speaker and speaker being held at chest height), at the three test frequency ranges (0-150Hz, 150-300Hz and 300-450Hz), and at three distances from the speaker (0cm, 85cm and 170cm). These data were accompanied by a negative control, measuring acceleration at all three test distances with no sound playing. Each treatment combination was measured in three separate trials.

These data were output as acceleration in x, y and z dimensions. The y dimension reflected vertical-aligned movement that related to floor vibrations. Measurement units were acceleration in units of gravities; we multiplied all measurements by 9.8 to convert to ms^-2^. We then cropped measurement intervals to the 2 seconds (60 measurements) in the centre of each measurement interval, to avoid edge effects when the sound was not played or when the technician was moving across the floor to start and stop the recording.

Given our intention to measure the magnitude of floor vibrations, we converted these measurements to absolute deviations from local gravitational acceleration with two steps. First, we averaged acceleration measurements in the negative control treatment. Actual gravity varies based on longitude, latitude and elevation, so we treated this average as “local gravity”. We subtracted this value from all measurements, creating a “deviation from local gravity” variable. We were only concerned with the magnitude of acceleration, not whether the phone was accelerating up or down. As such, we treated the absolute of these deviation measurements as the magnitude of acceleration. This results in an absolute deviation from local gravity measured in ms^-2^ at a 30Hz sampling rate.

Our test variables were not completely crossed when considering our negative control (which did not have foam treatment). We combined foam and frequency treatments together to obtain 10 aggregate categories (no sound plus all nine frequency x insulation combinations). We modelled absolute delta acceleration as a response variable (square root-transformed) as a function of these categories, interacting with distance (treated as a continuous variable), in a hierarchical Bayesian model. We nested each set of 60 measurements from each trial as a random effect. Coefficients from this model are shown in Table S6. Only two treatment combinations showed a significantly increased acceleration relative to the no sound control: the 0-150Hz frequency with or without foam.

We created a model without the distance and interaction effects to obtain mean and credible interval estimates for each treatment combination, which are shown in Figure S1.

To better understand the magnitude of vibrations from the significant treatments, we compared the 0-150Hz treatments with a position control, which were acceleration measurements obtained by a technician jumping on the floor immediately beside the phone. These are shown in Figure S2.

Table S1. Individual snakes included in the study, their size, age, foraging mode*, and temperament.

| **Snake name** | **Species** | **Common name** | **Sex** | **Total length**  **(m)** | **Age (years)** | **Foraging mode** |
| --- | --- | --- | --- | --- | --- | --- |
| Squishy | *Oxyuranus scutellatus* | Coastal Taipan | m | 2.2 | 22 | active |
| Boss | *Oxyuranus scutellatus* | Coastal Taipan | f | 1.8 | 14 | active |
| Fibonacci | *Oxyuranus scutellatus* | Coastal Taipan | m | 1.6 | 11 | active |
| Lower Jaw | *Oxyuranus scutellatus* | Coastal Taipan | f | 1.1 | 4 | active |
| Mawa | *Oxyuranus scutellatus* | Coastal Taipan | m | 1.1 | 4 | active |
| Lumpy | *Oxyuranus scutellatus* | Coastal Taipan | m | 1.4 | 11 | active |
| Upgrade | *Pseudonaja textilis* | Eastern Brown Snake | m | 1.8 | 12 | active |
| El Diablo | *Pseudonaja affinis* | Dugite | m | 1.2 | 15 | active |
| Mr Naughty | *Pseudonaja affinis* | Dugite | m | 1.2 | 15 | active |
| Perth Girl | *Acanthophis antarcticus* | Common Death Adder | f | 0.35 | 6 | ambush |
| Briggs | *Acanthophis antarcticus* | Common Death Adder | m | 0.44 | 8 | ambush |
| Glory | *Acanthophis antarcticus* | Common Death Adder | m | 0.4 | 6 | ambush |
| Dull Sister | *Acanthophis rugosus* | Rough-scaled Death Adder | f | 0.6 | 7 | ambush |
| Bright Sister | *Acanthophis rugosus* | Rough-scaled Death Adder | f | 0.6 | 7 | ambush |
| Bitey Boy | *Aspidites ramsayi* | Woma Python | m | 1.0 | 10 | active arboreal |
| Dorsal Girl | *Aspidites ramsayi* | Woma Python | f | 1.0 | 10 | active arboreal |
| Elapid Boy | *Aspidites ramsayi* | Woma Python | m | 0.9 | 8 | active arboreal |
| Casper | *Hoplocephalus bitorquatus* | Pale-headed Snake | m | 0.35 | 5 | arboreal |
| Chris' Hoplo | *Hoplocephalus bitorquatus* | Pale-headed Snake | m | 0.38 | 7 | arboreal |

*****[3–6]**.**

**Table S2**: PERMANOVA summary table testing whether dissimilarity of composition of defensive/cautious behaviors is clustered by genus, sound treatment, or their interaction.

|  | **DF** | **Sum of squares** | **R^2^** | **F** | **P** |
| --- | --- | --- | --- | --- | --- |
| Genus | 4 | 4.028 | 0.889 | 36.279 | 0.001 |
| Sound Factor | 1 | 0.163 | 0.036 | 5.884 | 0.001 |
| Genus: sound factor | 4 | 0.117 | 0.026 | 1.052 | 0.446 |
| Residual | 8 | 0.222 | 0.049 |  |  |
| Total | 17 | 4.530 | 1.000 |  |  |

Table S3-5 are Bayesian hierarchical model summary tables, along with additional model validation tests via residual simulation and leave-one-out cross validation (LOO). Each model is fit with the control treatment and the first genus alphabetically (*Aspidites* in Table S2 and *Acanthophis* is Tables S3-S4) as the global intercept. Estimates are means from Bayesian posteriors, along with standard errors (SE) and credible intervals (CI). Ȓ is a metric of model convergence, where 1 equals perfect convergence. ESS = effective sample size, a measure of the number of reliable samples obtained from the model markov chains. ELPD = theoretical expected log pointwise predictive density, a measure of model fit (can be read as per log-likelihood in frequentist models), P LOO = effective number of parameters, LOOIC = LOO information criterion. Pareto K is a measure of importance sampling reliability (where values < 0.5 are ideal).

**Table S3:** Probability of defensive/cautious behavior model summary table

| Fixed effects | Estimate | SE | Lower 95% CI | Upper 95% CI | Ȓ | Bulk ESS | Tail ESS |
| --- | --- | --- | --- | --- | --- | --- | --- |
| Intercept | 1.064 | 4.502 | -8.045 | 10.078 | 1.001 | 8145 | 10980 |
| soundFact1 | 1.537 | 1.039 | -0.451 | 3.638 | 1.000 | 8168 | 11984 |
| soundFact2 | 0.654 | 1.058 | -1.376 | 2.773 | 1.000 | 8446 | 12004 |
| soundFact3 | 1.696 | 1.064 | -0.324 | 3.859 | 1.000 | 7789 | 11268 |
| genusOxyuranus | -0.145 | 2.029 | -4.077 | 3.885 | 1.001 | 7853 | 11146 |
| genusPseudonaja | -0.341 | 2.355 | -5.002 | 4.317 | 1.000 | 7828 | 11639 |
| logAge | -0.960 | 1.801 | -4.577 | 2.675 | 1.001 | 7917 | 10736 |
| soundFact1:genusOxyuranus | -0.213 | 1.276 | -2.733 | 2.268 | 1.000 | 9087 | 12753 |
| soundFact2:genusOxyuranus | 1.281 | 1.292 | -1.229 | 3.815 | 1.000 | 9107 | 12791 |
| soundFact3:genusOxyuranus | -0.341 | 1.316 | -2.906 | 2.212 | 1.000 | 8600 | 13063 |
| soundFact1:genusPseudonaja | -1.279 | 1.493 | -4.263 | 1.650 | 1.000 | 10254 | 13393 |
| soundFact2:genusPseudonaja | -0.638 | 1.505 | -3.611 | 2.277 | 1.000 | 9731 | 13134 |
| soundFact3:genusPseudonaja | -1.024 | 1.510 | -3.957 | 1.908 | 1.000 | 9447 | 12556 |
|  |  |  |  |  |  |  |  |
| Random effects | Estimate | SE | Lower 95% CI | Upper 95% CI | Ȓ | Bulk ESS | Tail ESS |
| Speaker Side | 1.038 | 1.384 | 0.022 | 4.528 | 1.000 | 8431 | 10258 |
| Snake | 2.898 | 1.012 | 1.501 | 5.373 | 1.000 | 6950 | 11461 |
| Trial | 0.706 | 0.367 | 0.067 | 1.497 | 1.001 | 5559 | 5622 |
|  |  |  |  |  |  |  |  |
| Dispersion test | Obs:Sim | p-value |  |  |  |  |  |
|  | 0.935 | 0.408 |  |  |  |  |  |
|  |  |  |  |  |  |  |  |
| Uniformity test | D | p-value |  |  |  |  |  |
| One-sample Komogorov-Smirnov test | 0.039 | 0.918 |  |  |  |  |  |
|  |  |  |  |  |  |  |  |
| Leave-one-out cross validation | Estimate | SE |  |  |  |  |  |
| ELPD LOO | -112 | 8.7 |  |  |  |  |  |
| P LOO | 30.2 | 3.0 |  |  |  |  |  |
| LOOIC | 223.9 | 17.4 |  |  |  |  |  |
| Pareto K | 99.5% < 0.5 |  |  |  |  |  |  |

**Table S4:** Binary movement probability model summary

| Fixed effects | Estimate | SE | Lower 95% CI | Upper 95% CI | Ȓ | Bulk ESS | Tail ESS |
| --- | --- | --- | --- | --- | --- | --- | --- |
| Intercept | 4.601 | 3.194 | -1.607 | 11.021 | 1.000 | 8809 | 11155 |
| soundFact1 | 0.238 | 0.744 | -1.249 | 1.687 | 1.000 | 9090 | 13335 |
| soundFact2 | 0.246 | 0.746 | -1.228 | 1.703 | 1.000 | 9534 | 13815 |
| soundFact3 | -0.814 | 0.885 | -2.597 | 0.875 | 1.001 | 9240 | 12237 |
| genusAspidites | 1.778 | 1.683 | -1.592 | 5.056 | 1.000 | 8280 | 11625 |
| genusHoplocephalus | 2.226 | 1.968 | -1.635 | 6.152 | 1.000 | 10480 | 13554 |
| genusOxyuranus | 4.381 | 1.552 | 1.306 | 7.470 | 1.000 | 7726 | 11544 |
| genusPseudonaja | 2.965 | 1.936 | -1.087 | 6.622 | 1.000 | 7517 | 10972 |
| logAge | -3.120 | 1.472 | -6.060 | -0.222 | 1.000 | 9062 | 11803 |
| soundFact1:genusAspidites | 2.804 | 1.424 | 0.172 | 5.766 | 1.000 | 13036 | 14100 |
| soundFact2:genusAspidites | 2.276 | 1.343 | -0.285 | 4.983 | 1.000 | 12682 | 15313 |
| soundFact3:genusAspidites | 4.765 | 1.673 | 1.681 | 8.234 | 1.000 | 13673 | 14838 |
| soundFact1:genusHoplocephalus | 0.930 | 1.599 | -2.122 | 4.144 | 1.000 | 14270 | 15215 |
| soundFact2:genusHoplocephalus | -0.517 | 1.564 | -3.607 | 2.529 | 1.000 | 13501 | 14085 |
| soundFact3:genusHoplocephalus | 1.875 | 1.651 | -1.292 | 5.242 | 1.000 | 13246 | 14617 |
| soundFact1:genusOxyuranus | -0.243 | 1.156 | -2.523 | 2.053 | 1.000 | 12222 | 15156 |
| soundFact2:genusOxyuranus | 0.826 | 1.206 | -1.477 | 3.277 | 1.000 | 12353 | 13886 |
| soundFact3:genusOxyuranus | 0.806 | 1.253 | -1.601 | 3.311 | 1.001 | 11073 | 13058 |
| soundFact1:genusPseudonaja | -0.757 | 1.234 | -3.168 | 1.689 | 1.000 | 12400 | 14833 |
| soundFact2:genusPseudonaja | -0.755 | 1.286 | -3.296 | 1.749 | 1.000 | 12918 | 14222 |
| soundFact3:genusPseudonaja | 0.285 | 1.349 | -2.347 | 2.926 | 1.000 | 11746 | 14912 |
|  |  |  |  |  |  |  |  |
| Random effects | Estimate | SE | Lower 95% CI | Upper 95% CI | Ȓ | Bulk ESS | Tail ESS |
| Speaker Side | 0.913 | 1.312 | 0.018 | 4.278 | 1.000 | 7276 | 8774 |
| Snake | 2.026 | 0.577 | 1.146 | 3.373 | 1.001 | 7859 | 12714 |
| Trial | 0.226 | 0.176 | 0.008 | 0.649 | 1.000 | 10289 | 7965 |
|  |  |  |  |  |  |  |  |
| Dispersion test | Obs:Sim | p-value |  |  |  |  |  |
|  | 0.969 | 0.696 |  |  |  |  |  |
|  |  |  |  |  |  |  |  |
| Uniformity test | D | p-value |  |  |  |  |  |
| One-sample Komogorov-Smirnov test | 0.052 | 0.354 |  |  |  |  |  |
|  |  |  |  |  |  |  |  |
| Leave-one-out cross validation | Estimate | SE |  |  |  |  |  |
| Elpd LOO | -153.8 | 12.6 |  |  |  |  |  |
| P LOO | 37.7 | 4.2 |  |  |  |  |  |
| LOOIC | 307.7 | 25.1 |  |  |  |  |  |
| Pareto K | 97.5% <0.5 |  |  |  |  |  |  |

**Table S5:** Probability of movement towards speaker model summary

| Fixed effects | Estimate | SE | Lower 95% CI | Upper 95% CI | Ȓ | Bulk ESS | Tail ESS |
| --- | --- | --- | --- | --- | --- | --- | --- |
| Intercept | -0.170 | 1.626 | -3.225 | 2.938 | 1.001 | 6233 | 4587 |
| soundFact1 | 1.654 | 0.820 | 0.098 | 3.330 | 1.000 | 6224 | 9111 |
| soundFact2 | 1.708 | 0.823 | 0.140 | 3.394 | 1.000 | 6122 | 8898 |
| soundFact3 | 1.025 | 0.880 | -0.671 | 2.808 | 1.000 | 6845 | 10060 |
| genusAspidites | 2.034 | 0.996 | 0.116 | 4.014 | 1.000 | 6787 | 10889 |
| genusHoplocephalus | 0.984 | 1.142 | -1.298 | 3.223 | 1.000 | 7606 | 11205 |
| genusOxyuranus | 0.564 | 0.980 | -1.383 | 2.497 | 1.000 | 6434 | 9128 |
| genusPseudonaja | 0.383 | 1.351 | -2.479 | 2.875 | 1.000 | 7669 | 9554 |
| logAge | -1.242 | 0.500 | -2.261 | -0.287 | 1.001 | 13219 | 11028 |
| soundFact1:genusAspidites | -0.891 | 1.154 | -3.149 | 1.362 | 1.000 | 8313 | 11172 |
| soundFact2:genusAspidites | -2.145 | 1.225 | -4.591 | 0.215 | 1.000 | 8392 | 11890 |
| soundFact3:genusAspidites | -2.840 | 1.573 | -6.232 | -0.032 | 1.000 | 11231 | 13246 |
| soundFact1:genusHoplocephalus | -1.060 | 1.365 | -3.691 | 1.635 | 1.000 | 9342 | 12356 |
| soundFact2:genusHoplocephalus | -1.742 | 1.430 | -4.603 | 1.002 | 1.000 | 9184 | 13150 |
| soundFact3:genusHoplocephalus | -0.164 | 1.404 | -2.885 | 2.602 | 1.000 | 9091 | 12574 |
| soundFact1:genusOxyuranus | -0.451 | 1.137 | -2.706 | 1.807 | 1.000 | 7566 | 11388 |
| soundFact2:genusOxyuranus | -0.109 | 1.110 | -2.291 | 2.073 | 1.000 | 6975 | 9831 |
| soundFact3:genusOxyuranus | 0.295 | 1.173 | -1.989 | 2.617 | 1.000 | 7873 | 11060 |
| soundFact1:genusPseudonaja | -0.609 | 1.555 | -3.535 | 2.571 | 1.000 | 9342 | 10563 |
| soundFact2:genusPseudonaja | 0.166 | 1.507 | -2.699 | 3.224 | 1.000 | 8906 | 10827 |
| soundFact3:genusPseudonaja | -0.893 | 1.791 | -4.480 | 2.542 | 1.000 | 10036 | 12216 |
|  |  |  |  |  |  |  |  |
| Random effects | Estimate | SE | Lower 95% CI | Upper 95% CI | Ȓ | Bulk ESS | Tail ESS |
| Speaker Side | 1.008 | 1.415 | 0.023 | 4.695 | 1.001 | 5738 | 5104 |
| Snake | 0.406 | 0.286 | 0.018 | 1.073 | 1.001 | 5838 | 8223 |
| Trial | 0.335 | 0.231 | 0.016 | 0.869 | 1.000 | 6750 | 8732 |
|  |  |  |  |  |  |  |  |
| Dispersion test | Obs:Sim | p-value |  |  |  |  |  |
|  | 1.008 | 0.912 |  |  |  |  |  |
|  |  |  |  |  |  |  |  |
| Uniformity test | D | p-value |  |  |  |  |  |
| One-sample Komogorov-Smirnov test | 0.029 | 0.955 |  |  |  |  |  |
|  |  |  |  |  |  |  |  |
| Leave-one-out cross validation | Estimate | SE |  |  |  |  |  |
| Elpd LOO | -186.5 | 11.7 |  |  |  |  |  |
| P LOO | 33.9 | 3.2 |  |  |  |  |  |
| LOOIC | 372.9 | 23.4 |  |  |  |  |  |
| Pareto K | 99.1% <0.5 |  |  |  |  |  |  |

**Table S6:** Ground vibration acceleration model summary

| Fixed effects | Estimate | SE | Lower 95% CI | Upper 95% CI | Ȓ | Bulk ESS | Tail ESS |
| --- | --- | --- | --- | --- | --- | --- | --- |
| Intercept (negative control) | 0.068 | 0.006 | 0.056 | 0.081 | 1.036 | 144.124 | 242.289 |
| **0-150HzFoam** | **0.096** | **0.009** | **0.079** | **0.113** | **1.022** | **196.215** | **292.722** |
| 0-150HzHeld | 0.015 | 0.008 | -0.002 | 0.031 | 1.019 | 232.045 | 407.782 |
| **0-150HzNoInsulation** | **0.086** | **0.009** | **0.069** | **0.104** | **1.031** | **206.833** | **362.045** |
| 150-300HzFoam | 0.006 | 0.009 | -0.010 | 0.023 | 1.021 | 185.682 | 231.100 |
| 150-300HzHeld | -0.009 | 0.008 | -0.024 | 0.008 | 1.030 | 162.029 | 523.757 |
| 150-300HzNoInsulation | 0.000 | 0.008 | -0.016 | 0.017 | 1.027 | 216.301 | 364.369 |
| 300-450HzFoam | 0.007 | 0.008 | -0.008 | 0.024 | 1.033 | 164.850 | 414.623 |
| 300-450HzHeld | -0.004 | 0.008 | -0.021 | 0.013 | 1.022 | 251.284 | 411.078 |
| 300-450HzNoInsulation | -0.006 | 0.008 | -0.023 | 0.010 | 1.023 | 213.055 | 392.739 |
| Distance from speaker | -2.223E-05 | 5.923E-05 | -1.373E-04 | 9.340E-05 | 1.026 | 235.942 | 607.221 |
| 0-150HzFoam:Distance | -4.817E-05 | 7.759E-05 | -2.021E-04 | 9.874E-05 | 1.014 | 306.462 | 655.615 |
| 0-150HzHeld:Distance | 3.929E-05 | 7.771E-05 | -1.145E-04 | 1.901E-04 | 1.018 | 339.530 | 592.873 |
| 0-150HzNoInsulation:Distance | 6.625E-05 | 7.962E-05 | -8.571E-05 | 2.325E-04 | 1.022 | 277.988 | 464.487 |
| 150-300HzFoam:Distance | -3.076E-08 | 7.786E-05 | -1.549E-04 | 1.537E-04 | 1.012 | 304.729 | 753.008 |
| 150-300HzHeld:Distance | 5.980E-05 | 7.513E-05 | -9.087E-05 | 2.050E-04 | 1.020 | 280.482 | 894.499 |
| 150-300HzNoInsulation:Distance | 4.431E-05 | 7.747E-05 | -1.031E-04 | 1.969E-04 | 1.026 | 306.532 | 746.601 |
| 300-450HzFoam:Distance | -4.595E-05 | 7.449E-05 | -1.944E-04 | 9.826E-05 | 1.020 | 305.144 | 1085.318 |
| 300-450HzHeld:Distance | 4.433E-05 | 7.723E-05 | -1.155E-04 | 1.963E-04 | 1.020 | 351.307 | 786.411 |
| 300-450HzNoInsulation:Distance | 1.749E-05 | 7.492E-05 | -1.288E-04 | 1.671E-04 | 1.017 | 416.981 | 717.801 |
|  |  |  |  |  |  |  |  |
| Random effects | Estimate | SE | Lower 95% CI | Upper 95% CI | Ȓ | Bulk ESS | Tail ESS |
| Trial grouping | 0.008 | 0.001 | 0.007 | 0.011 | 1.001 | 1444.739 | 2262.327 |
|  |  |  |  |  |  |  |  |
|  |  |  |  |  |  |  |  |
| Uniformity test | D | p-value |  |  |  |  |  |
| One-sample Komogorov-Smirnov test | 0.041 | 3.709E-8 |  |  |  |  |  |
|  |  |  |  |  |  |  |  |
| Leave-one-out cross validation | Estimate | SE |  |  |  |  |  |
| Elpd LOO | 9417.3 | 75.4 |  |  |  |  |  |
| P LOO | 70.9 | 2.1 |  |  |  |  |  |
| LOOIC | -18834.6 | 150.7 |  |  |  |  |  |
| Pareto K | 97.5% <0.5 |  |  |  |  |  |  |

Movie S1.

[Here](https://youtu.be/jnNzinwwItk) is a 2-minute video containing a representative example of each snake behaviour: <https://youtu.be/jnNzinwwItk>

1. Viecelli C, Graf D, Aguayo D, Hafen E, Füchslin RM. Using smartphone accelerometer data to obtain scientific mechanical-biological descriptors of resistance exercise training. PLoS One. 2020;15. doi:10.1371/journal.pone.0235156

2. Mourcou Q, Fleury A, Franco C, Klopcic F, Vuillerme N. Performance evaluation of smartphone inertial sensors measurement for range of motion. Sensors. 2015;15: 23168–23187. doi:10.3390/s150923168

3. Bruton MJ. Arboreality, excavation, and active foraging: Novel observations of radiotracked woma pythons *Aspidites ramsayi*. Mem Queensl Museum - Nat. 2013;56: 313–329.

4. Shine R. Australian snakes: A natural history. Singapore: Imago Productions; 1991.

5. Shine R. Arboreality in snakes: Ecology of the Australian Elapid genus *Hoplocephalus*. Copeia. 1983;1983: 198. doi:10.2307/1444714

6. Hagman M, Phillips BL, Shine R. Tails of enticement: Caudal luring by an ambush-foraging snake (*Acanthophis praelongus*, Elapidae). Funct Ecol. 2008;22: 1134–1139. doi:10.1111/j.1365-2435.2008.01466.x
